# Biodecomposition with *Phanerochaete chrysosporium*: A review

**DOI:** 10.3934/microbiol.2024046

**Published:** 2024-11-25

**Authors:** Delon Konan, Adama Ndao, Ekoun Koffi, Saïd Elkoun, Mathieu Robert, Denis Rodrigue, Kokou Adjallé

**Affiliations:** 1 Laboratory of Environmental Biotechnologies, Institut National de la Recherche Scientifique (INRS), Québec city, QC, G1P 4S5, Canada; 2 Department of Mechanic and Energy Engineering, Institut National Polytechnique Felix Houphouët Boigny (INPHB), Yamoussoukro, Côte d'Ivoire; 3 Center for Innovation in Technological Ecodesign (CITE), University of Sherbrooke, Sherbrooke, QC, J1K 2R1, Canada; 4 Department of Chemical Engineering, Université Laval, Québec City, QC, G1V0A6, Canada

**Keywords:** *Phanerochaete chrysosporium*, biodecomposition, lignocellulosic biomass, lignin, xenobiotic aromatic compounds

## Abstract

*Phanerochaete chrysosporium* is considered the model fungus for white rot fungi. It is the first basidiomycete whose genome has been completely sequenced. Its importance lies in the fact that its enzymatic system comprises the major enzymes involved in lignin degradation. Lignin is a complex and highly recalcitrant compound that very few living organisms are capable of degrading naturally. On the other hand, the enzymes produced by *P. chrysosporium* are also powerful agents for the mineralization into CO_2_ and H_2_O of a wide range of aromatic compounds. However, these aromatic compounds are largely xenobiotic compounds with documented toxic effects on the environment and health. While the economic and environmental benefits of biodegradation with *P. chrysosporium* are well established, a thorough understanding of *P. chrysosporium* and its biodegradation processes is essential for successful biodegradation. Our aim of this critical literature review is to provide a concise and comprehensive insight of biodecomposition of organic substrate by *P. chrysosporium*.

## 1. Introduction

Biodecomposition, biodegradation, or biological decomposition are all the processes, procedures, and means that lead to the degradation of a compound using microorganisms (bacteria, yeast, fungi, etc.) [1]. Biodecomposition is a field of expertise in its own, with its theories, methods, concepts, techniques, and tools. Its importance in research has grown with the development of industries. The development of this field is closely linked to that of industrial production. Indeed, the exploitation of industries and their diversification over the years has given rise to several major environmental problems, including water pollution [2],[3]. Industrial effluents and manufactured products are heavily laden with pollutants of many types. These pollutants find their way into natural environments and cause significant damage to flora and fauna; hence, the need to treat these effluents before they are discharged into the environment. Certain phenolic and aromatic compounds are persistent xenobiotic pollutants with serious consequences for the health of living organisms, including humans [4]. They are present in effluents of a variety of industries (pulp and paper, agrifood, textiles, pharmaceuticals, chemicals, ceramics, electronics, etc.). Their elimination or degradation has been the subject of several chemical wastewater pretreatment methods. However, the cost, ecological aspect and effectiveness of these chemical methods pose problems. Biodecomposition, as a wastewater treatment method, has been developed based on the ability of certain microorganisms to degrade, decompose and metabolize phenolic compounds. The most promising of these microorganisms are fungi, especially white rot fungi (WRF). They are naturally equipped to degrade phenolic compounds through a system of intra- and extracellular enzymes [5]. These organisms can thrive in both liquid and solid media. That also contributes to their suitability as site decontamination agents [6]. They also have the reputation of being the most efficient organisms for the degradation of one of the most complex compounds in the living world: Lignin. Lignin is one of the major components of lignocellulosic biomasses [7]. It is the main obstacle to the development of second-generation biorefineries, which aim to convert the cellulose and hemicellulose contained in lignocellulosic biomasses into biofuels. Today, lignin is the focus of much attention in biotechnology and materials science. The reasons for this situation are manifold. At a socio-economical level, petroleum-derived products are increasingly the subject of bad press because of their environmental cost. Despite the petrochemical industry's efforts to green its reputation, the balance is increasingly tipped in the direction of a definitive and urgent move away from non-renewables. Yet, lignocellulosic biomass is the only renewable source of energy that is most similar in composition and application to fossil fuels (oil, coal, etc.) [8]. Moreover, this type of biomass is the first and largest reservoir of cellulose, a compound highly prized in the materials and biorefinery sectors [9]. Because of their unique ability to break down lignin and aromatic compounds, fungi, especially white rot fungi (WRF), are used in the treatment of lignocellulosic biomasses, industrial effluents, for decontamination, bioremediation, etc. *Phanerochaete chrysosporium* is by far the most popular WRF because its many applications. The aim of this literature review is to provide the reader with a fundamental and in-depth understanding of this fungi in the context of biodegradation, by investigating the latest knowledge on the subject.

## 2. Classification in the fungi kingdom

*P. chrysosporium* is a fungus of the phylum Basidiomycetes, of the class Agaricomycetes, of the Polyporales order, and of the Phanerochaetaceae family. Basidiomycetes are the second most numerous phylum in the fungi kingdom, after Ascomycetes. They are estimated to contain over 40,000 different species of fungi [10]. Basidiomycetes are mainly characterized by the shape of their spore-producing organs, called basidia, which are reminiscent of a club or club-like shape with spores at the ends. In some species of basidiomycetes organized in hyphae, the hyphae is septate, i.e. the nuclei of the hyphae are separated from each other by septa [11]. Basidiomycetes are made up of three (or four, depending on the author) classes, the broadest of which is the Agaricomycetes. Most Agaricomycetes are saprophytic fungi [12]. They are found in environments rich in organic matter. They play a crucial role in ecosystems, mineralizing nutrients and making them available to other growing plant and animal organisms. This group includes white and brown rot fungi. They are also pathogens when they target living organisms. The Agaricomycetes are highly diversified in terms of morphology and also include a large proportion of well-known edible fungi such as the popular commercial mushroom (*Agaricus bisporus*), the oyster mushroom (*Pleutorus oestratus*), and the Shiitake (*Lentinula edodes*) [13]. The Polyporales order of Agaricomycetes essentially contains ligninolytic fungi (capable of degrading lignin). Some fungi in this order can parasitize the roots of certain plants or form mycorrhizal associations with orchids. Polyporales are frequently isolated from woody tissues and plant roots (endophytes) [14]. As for the Phanerochaetaceae family, it is frequently subject to rearrangements or additions of genera based on new species discoveries or phylogenetic reclassification of existing ones [15]. However, the *Phanerochaete* genus in the Phanerochaetaceae family is stable. It groups together fungi that cause white rot on wood. Their fruiting structure is a monomitic septate hypha (composed of a single type of hypha). To date, the international Catalogue Of Life (COL) database contains 105 species of Phanerochaetes. Few data exist in the literature on the *Phanerochaete* genus, but one of its species is the focus of much attention. This is *P. chrysosporium*. Other well-known Phanerochaetes include *Phanerochaete velutina* for bioremediation, and *Phanerochaete sordida* for biotransformation [16],[17].

## 3. Genetic identity

The *P. chrysosporium* genome was the first of the white rot fungi (and basidiomycetes in general) to be completely sequenced. The number of chromosomes can vary considerably from one strain to another (e.g., 10 chromosomes for *P. chrysosporium* BKMF-1767 and 11 for *P. chrysosporium* ME-446 [18], but the genome is composed of almost 30 million base pairs. Today, research is focused on identifying the functions of the constituent genes [19],[20]. Genes encoding the production of lignolytic enzymes have a prominent place in this research. The lignolytic enzymes of interest are manganese peroxidase, laccase, lignin peroxidase, and the cytochrome P450 family. By analyzing the genes responsible for manganese peroxidase (MnP) production, Kuppuraj et al. [21] identified five (5) different manganese peroxidases produced by *P. chrysosporium*, ranging in size from 32 to 62 kDa and consisting of around 380 amino acids each. The genes encoding these enzymes comprise short chains of 6 or 7 introns [22]. Expression of these genes occurs late in the life cycle, practically during the mature phase of the fungi. Moreover, manganese peroxidase secretion is triggered only under conditions of thermal stress and when the environment contains specific cofactors such as MnO_2_ and hydrogen peroxide (H_2_O_2_). The quantity and quality of enzymes produced depend on these exogenous factors. According to Emami et al (2020), heterogeneous iron oxide (Fe_2_O_3_) (containing alginate) significantly boosts MnP production by almost 70%.

The production of laccase in *P. chrysosporium* is highly controversial. Indeed, analysis of the *P. chrysosporium* genome has failed to reveal any genome regions coding for laccases known to date [23]. However, some studies have demonstrated laccase production by *P. chrysosporium* [24],[25]. Indeed, in the 90s, it was unclear whether *P. chrysosporium* production was dependent on environmental factors, or whether *P. chrysosporium* was genetically deficient in laccase production [26],[27]. To date, although there is very little information concerning the genes responsible for laccase production in *P. chrysosporium*, it is nevertheless accepted that, like MnPs, laccase is produced as a result of environmental conditions and copper (Cu^2+^)-containing inducers such as copper sulfate [28]. Laccase production has been observed in the presence of high levels of nitrogen, glucose and copper [29]. Production can be even higher when co-cultured with *Trametes versicolor* or in the presence of manganese IV oxide [25],[26]. The addition of veratryl alcohol, 2,5-xylidine, fructose (more than 100-fold in certain basidiomycetes), glucose, and cellobiose to the culture medium has been reported to significantly increase laccase production [30]. Laccase is reported to play the dual role of depolymerizing lignin and repolymerizing quinones and phenols (products of lignin degradation), with the aim of preserving fungi from these compounds which, once released into the environment, become toxic to fungi [26]. The production of laccase in white rot fungi would therefore appear to be a defense mechanism resulting from adaptation. However, studies have demonstrated the ability of recombinant strains of *P. chrysosporium* to produce laccase using genes from other white rot fungi strains well known for laccase production, such as *Trametes versicolor* and *Pleutorus erynngii* [23],[31]. Laccase transcription has been reported to be mediated by different genes, but gene sequences range from 515 to 619 amino acids.

The lignin peroxidase family comprises several isoenzymes. These enzymes are synthesized from clustered genes separated by 8 or 9 short intron chains [22]. Each isoenzyme is encoded by a different gene of 343 to 344 amino acids. The genes encoding these enzymes have been located on 5 of the 10 chromosomes of *P. chrysosporium* BKMF-1767 and on 4 of the 11 chromosomes of *P. chrysosporium* ME-446. Like manganese peroxidases and laccases, LiP expression is influenced by the nutrient composition of the medium. Excess nitrogen and carbon in the medium leads to non-secretion of LiP and MnP, while excess Mn(II) boosts MnP production but suppresses LiP expression [18],[32]. A total of 154 genes encoding P450somes have been identified in the *P. chrysosporium* genome, constituting around 1% of the genome [19]. Research into cytochromes P450s (CYPs) is more recent than the other three enzyme types discussed. Furthermore, cytochromes are secondary metabolites. They are a family of enzymes produced by many microorganisms, including human cells. They play several roles, such as elimination of toxic substances, and are involved in fatty acid metabolism in human cells [33]. In *P. chrysosporium*, they are also involved in the degradation of phenolic compounds [34],[35]. Analysis of the *P. chrysosporium* genome has identified over 150 genes encoding P450somes, covering more than 12 families and 23 subfamilies. This is one of the largest numbers of genes encoding P450somes ever identified in a fungal genome. The cytochrome P450 family includes CYP51, CYP53, CYP61, CYP63, CYP505, CYP5158A1, and CYP5144C8 [36]–[38]. All 150 genes have been shown to be expressed regardless of the conditions under which *P. chrysosporium* is grown: Lignolitic (low nitrogen content) or non-lignolitic (high nitrogen content). However, 23 of these genes are upregulated in the presence of high nitrogen levels and 4 are upregulated in the opposite case, i.e., when there is a low level of nitrogen [39].

Since the earliest days of the research into the genetic identity of *P. chrysosporium* in the 1980s, researchers have been interested in gene cloning, using a variety of techniques available at the time. In 1989, Margaret et al. [40] successfully carried out the first genetic transformation of an adenine auxotrophic strain of *P. chrysosporium*. This involved introducing into the DNA sequence of the adenine deficient *Phanerochaete* strain, the corresponding ade2 gene isolated from another fungus strain (*Schizophyllum commune*). After this study, several others followed with genetic transformations involving several other genes. In 1998, the genetic transformation of *P. chrysosporium* took another step forward with the introduction of the λYES expression vector, enabling the construction of a complementary DNA library for *P. chrysosporium* [41]. When the genome of *P. chrysosporium* was finally completely sequenced in 2004 by Martinez et al. [20], in 2006 Sharma et al. [42] proposed a less costly and rather efficient approach to genetic transformation of *P. chrysosporium*. This approach, used in other contexts with other microorganisms, involved co-culturing pellets of the auxotrophic strain with pellets of the virulent strain containing the gene to be cloned. In 2012, Suzuki et al. [43]'s comparative study of the genomes of *P. chrysosporium* and P. chrysosporium provided a better understanding of the genome of *P. chrysosporium*, and the genetic reasons for their natural tendency to colonize hardwood more than softwood. While gene cloning in *P. chrysosporium* continues to be the subject of research in recent years, primacy is given to genes encoding enzymes of the ligninolytic enzyme system, certainly because of the potential of these enzymes for various cutting-edge applications and their future economic value.

## 4. Natural habitat

While some studies restrict the presence of *P. chrysosporium* to North America and Africa, the fact is that it can be found in woodland and temperate ecosystems throughout the world [44]. It proliferates particularly on dead wood (branches, stems, leaves, etc.), but also evolves in various soil and water environments and even on animals. For example, Vivekanandhan et al. [45], Lui et al. [46], Dahiya et al. [47], and Zhao et al. [48] isolated it in India from the effluent of a sago food processing plant, in China from mountain trees, in Canada from the soil of a sugar factory landfill, and in South Africa from an indigenous forest. In general, *P. chrysosporium* prefers humid environments with temperatures close to 40 °C.

## 5. Isolation technique and natural substrate

**Figure 1.**
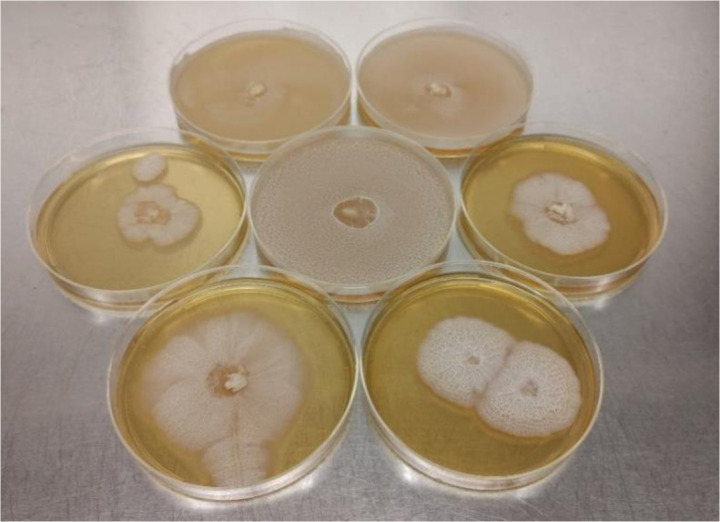
*P. chrysosporium* on petri dish started with a piece of mycelium as inoculum. Incubation temperature was 30 °C and culture media was Yeast Broth BD 271120.

Most of the *P. chrysosporium* strains used in research come from biotechnology companies, associations, or institutes such as the American Type Culture Collection (ATCC) in the USA, the Deutsche Sammlung von Mikroorganismen und Zellkulturen (DSMZ) in Germany and the National Collection of Yeast Cultures (NCYC) in the UK. The advantage of using strains from biotechnology organizations is the time saved and the reliability of the Phanerochaete strain used. This is particularly useful in research to compare results with those in the literature. In addition, these companies offer genetically modified strains of *P. chrysosporium* for specialized applications. To date, several different strains of *P. chrysosporium* are commercially available and can be identified by an alphanumeric code following the species name. For example, *P. chrysosporium* B-22 is effective for parasitizing nematode species [49]. However, for one reason or another, it may be useful to isolate one or more strains of *P. chrysosporium* from its natural environment. In this case, isolation involves a series of steps that can be summarized in four consecutive stages: (i) In-situ sampling of a portion of the natural environment, (ii) inoculation onto a suitable synthetic medium, (iii) strain identification, and (iv) subculture of the desired strain. The first step consists in identifying an environment suited to the development of the desired strain, and a time of year when its activity is likely to be at its peak. This could be, for example, damp dead wood in a state of advanced decomposition during hot periods of the year; or damp soil covered by a high concentration of woody debris [50]. Better still, if fructification of the strain is visible, the first step can be completed by directly sampling the carpophore with a portion of its medium to recover the underlying mycelium. The sampling step should preferably be conducted in a sterile tube or container, to avoid external contamination of the sample and make the third step less difficult. The culture medium may or may not be selective but should contain at least the nutrients required for strain development (see section 8). The agar culture is then incubated at 25 to 40 °C for 48 to 72 hours to allow sufficient time for the microorganism species to develop. Once colonies and mycelia are clearly visible, identification can begin. This is a tedious and sometimes frustrating stage. It consists of morphological identification [48],[51]. *P. chrysosporium* presents different morphologies depending on the environment and conditions in which it is found. When grown in petri dishes, it develops a beautiful, white, filamentous radial mycelium that is densely reticulated after 48 hours (Figure 1). Under certain conditions, not yet fully understood, small tufts of fluffy, whitish cotton-like fuzz form on the mycelium. On dead wood, *P. chrysosporium* develops a mycelium with the appearance of a smooth and uniform concretion of plank paint [52]. Open-access online databases are available to help identify fungus species. These databases provide information on each species listed, with supporting photos for some of them. Among the best known are Mushroom Observer, MycoBank, Catalogue of Life and Index Fungorum [53]. However, *P. chrysosporium* can easily be confused with *Phanerochaete velutina*, *Phanerochaete sordida*, *Phanerochaete laevis*, *Phanerochaete tuberculata*, and *Phanerochaete bubalina*, as their appearance are very similar. For this reason, in addition to morphological evaluation, further identification may be required through molecular and biochemical analyses targeting DNA. Franco-Duarte et al. [54] present a useful overview of the essentials of these analytical techniques. Once the *Phanerochaete* strain has been identified, the next step is the cultivation of the organism in liquid medium or on agar for 24 to 48 hours. Samples of the liquid culture broth or strain mycelium (on agar) can then be stored at −4 °C for future use.

**Figure 2.**
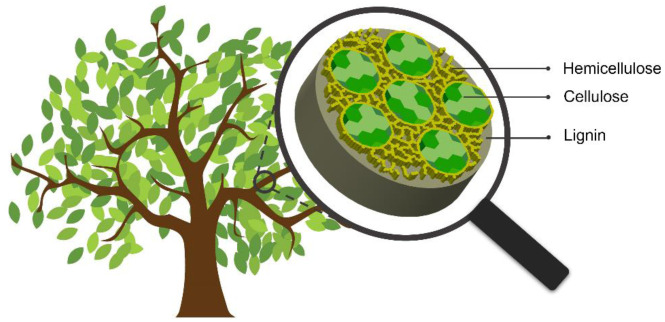
Structure of a lignocellulose microfiber.

Lignin is the natural substratum of *P. chrysosporium*, which it degrades to find carbon sources and food. Lignin is the most abundant natural aromatic polymer on earth [55]. It is found in plant cell walls, where it plays a structural role, as well as a protective role against physical, chemical and biological aggressions external to the plant [56]. Lignin is extremely ramified and is composed of three monomers: p-coumaryl alcohol, coniferyl alcohol, and sinapyl alcohol. Its chemical structure is highly irregular, differs within the same species, and depends on factors such as: The plant's stage of development, environmental conditions, and the part of the plant concerned (trunk, stem, straw, branch, etc.). These factors exert a major influence on the monomeric composition of lignin and affect its structure. Lignin is the second main constituent of lignocellulosic biomasses (wood and agricultural residues) after cellulose and before hemicellulose. In lignocellulose microfibers, i.e., lignin acts as a binder and barrier to cellulose and hemicellulose (Figure 2) [57]. This is a major obstacle to the conversion of lignocellulosic biomasses into biofuels by recovering the carbohydrates (cellulose and hemicellulose) contained in these biomasses. Despite lignin's complex structure, white rot fungi such as *P. chrysosporium* have acquired the ability to degrade it, its derivatives and a wide range of aromatic compounds, including pesticides, environmental pollutants, dyes and toxic waste [58]. They achieve this through a complex system of extracellular enzymes they secrete. It is this particular ability of WRFs to degrade such complex compounds that is attracting research attention. As for *P. chrysosporium*, it is considered the model WRF [59]. It possesses one of the most complete enzyme systems, encompassing the major lignolytic enzymes known to date. This gives it a significant efficiency in the degradation of woody and aromatic compounds. Lignin degradation leaves cellulose virtually intact in lignocellulosic biomass and gives the white color to wood degraded by *P. chrysosporium*.

## 6. Enzymatic system

Most white rot fungi can degrade lignin and lignin-like compounds using a non-substrate specific enzyme system. This system generally consists of one or two enzyme groups. Only a handful of species can secrete a complete ligninolytic enzyme system, that is, the three key groups namely laccases, manganese peroxidases and lignin peroxidases. *P. chrysosporium* and *Trametes versicolor* are two such species. However, the former remains one of the most widely used fungi, certainly because it was one of the first to be studied in the laboratory, and because the complete genome sequence is now available.

### 6.1. Key enzyme

*P. chrysosporium* is a heterotrophic organism that obtains its nutrients from the decomposition of woody plant matter and aromatic compounds in its environment. When nutrients are not directly available, extracellular enzymes are secreted to degrade these compounds. Only a handful of microorganisms are capable of this task. These are essentially fungi. They are grouped together under the name of rot fungi. There are three main types: brown rot fungi (BRF), soft rot fungi (SRF) and white rot fungi (WRF), in reference to their respective color/aspect on the wood they colonize. SRF degrade cellulose and leave lignin untouched, while WRF degrade lignin and leave cellulose intact. SRFs do the same as WRFs, degrading lignin and leaving only carbohydrates, but do so less efficiently in terms of degradation yield [60]. The lignolytic enzyme system of *P. chrysosporium* is recognized as the most complete of all enzyme complexes produced by other lignolytic fungi. This is one of the main reasons why *P. chrysosporium* is considered the model strain for lignolytic activity [61]. *P. chrysosporium* has a system composed of three main enzymes that work in concert to degrade lignin, phenolics, aromatics and similar compounds. These are laccase, lignin peroxidase, and manganese peroxidase, which are oxidoreductases. Apart from these enzymes, *P. chrysosporium* also produces several other enzymes such as methanol oxidase, 1,4-benzoquinone reductase, methyltransferase and cytochrome P450s [62]. Some enzymes of the P450 family are thought to be involved in the xenobiotic activity of *P. chrysosporium* through O-demethylation at the ring break of aromatic compounds such as Methoxyhydroquinone (MHQ) [19].

### 6.2. Manganese peroxidases

**Figure 3.**
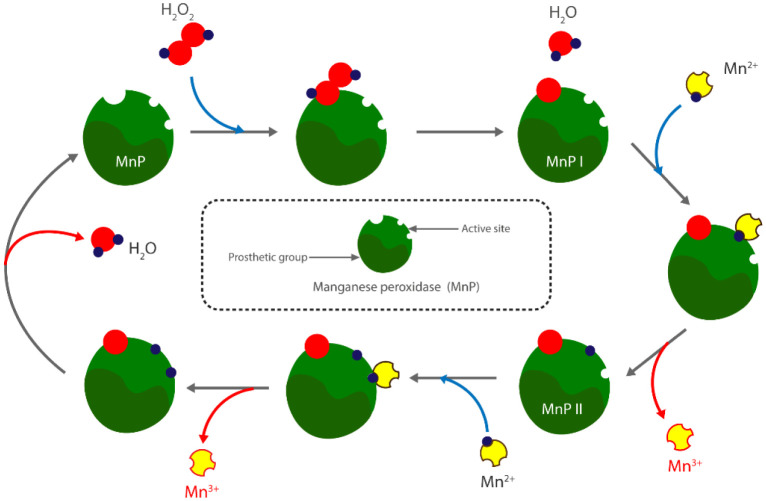
Transformation mechanism of Mn^2+^ into Mn^3+^ by manganese peroxidases.

MnPs (EC 1.11.1.13) are a group of enzymes using Mn^2+^ as a substrate. This enzyme was first purified and characterized by Jeffrey K. Glen and Michael in 1985 from *P. chrysosporium* [63]. They are acid glycoproteins with ph 4.5 and a molecular weight of around 45 kDa. MnPs are hemoproteins, i.e., they consist of two parts: (i) A protein part and (ii) a prosthetic group, the heme containing the iron cofactor (Fe^3+^). The heme is the enzyme's active site. There are more than a dozen MnPs. Their role is to oxidize Mn^2+^ to Mn^3+^. This catalysis takes place in three stages, according to reactions Eqs (1–3). Figure 3 illustrates the transformation mechanism. The MnP enzyme binds with hydrogen peroxide (or other peroxide) at the heme to form a first complex. In this complex, the iron of the heme enters into a bond with one of the oxygen atoms of the peroxide. The oxygen-oxygen bond in the peroxide is then cleaved by the transfer of two electrons from the heme to the peroxide. This results in the production of a water molecule and a Fe^4+^-oxo-porphirin radical called MnP I complex. The MnP I complex in turn oxidizes Mn^2+^ to Mn^3+^. This electron gain leads to a second radical compound, MnP II, which in turn oxidizes another Mn^2+^ ion to Mn^3+^. This second electron obtained by MnP II during this operation enables the release of the oxygen atom attached to the heme, with the formation of a water molecule, a return to the initial enzyme and a second Mn^3+^ ion. In this way, MnP enables one molecule of peroxide and two Mn^2+^ ions to be converted into 2 molecules of water and 2 Mn^3+^ ions [63]–[65].

$$\text{MnP}+{\text{H}}_{2}{\text{O}}_{2}\to \text{MnP I}+{\text{H}}_{2}\text{O}$$ (1)

$$\text{MnP I}+Mn^{2+}\to \text{MnP II}+Mn^{3+}$$ (2)

$$\text{MnP II}+Mn^{2+}\to \text{MnP}+Mn^{3+}+{\text{H}}_{2}\text{O}$$ (3)

Mn^3+^ ions are highly reactive. They react rapidly with chelating agents produced by the fungus such as oxalic acid to stabilize. The resulting low-molecular-weight compound is a powerful redox mediator capable of radicalizing the phenolic structures of lignin. The radicals created in the lignin structure trigger the spontaneous disintegration of lignin. The principle of degradation remains broadly the same with phenolic compounds other than lignin. MnPs are sensitive to peroxide concentration. Too much peroxide leads to inactivation of the enzyme [66].

### 6.3. Lignin peroxidases

LiPs isoenzymes are globular or helical glycoproteins of 40 kDa, consisting of 343 or 344 amino acids and typically fit into a volume of 50 x 40 x 40 Å [67]. LiPs (EC 1.11.1.14) are also heme enzymes. They have an optimal pH of 3. They feature among the highest redox potentials of peroxidases (Eo = 1.2 V at Ph 3); significantly higher than MnPs and Laccases, both of which display 0.8 V at their optimal pH (ph 4.5 and ph 5.5, respectively) [68]. This property confers on LiP the ability to catalyze the oxidation of non-phenolic aromatic compounds even in the absence of mediators. Two calcium ions (Ca^2+^) are present in their structure, as well as numerous N- and O-glycosylation sites. The LiP heme is embedded within the protein, with a narrow opening to the external environment. The default substrate for LiP is the 3,4-dimethoxylbenzyl alcohol known as veratryl alcohol. Like hydrogen peroxide, veratryl alcohol is a secondary metabolite produced by *P. chrysosporium*. The reaction chain produced by LiP is similar to that of MnP, except that instead of Mn, LiP uses veratryl alcohol as a substrate. It begins with the oxidation of Fe^3+^ from heme to form the first LiP I complex, with the production of a water molecule. LiP I is an oxo-ferryl intermediate deficient in the two electrons it has surrendered to the peroxide to form the water molecule. LiP I is then transformed into a second intermediate LiP II by reaction with veratryl alcohol, which acts as an electron donor. The LiP II compound is therefore electron-deficient. The cycle is completed when another veratryl alcohol molecule reacts with LiP II to yield an electron [68]. The enzyme returns to its initial state (LiP) and the cycle can resume as many times, as long as substrates are available and ph and temperature conditions are favorable. Eqs (4–6) summarize the reactions. The intermediate compounds LiP I and LiP II are highly reactive forms of LiP [69]. Apart from veratryl alcohol, they can react with numerous other phenolic and non-phenolic compounds, including lignin monomers and their derivatives (guaiacol, syringic acid, catechol, vanillyl alcohol, etc.), attacking β-O-4, C-C, C-O-C bonds and side chains. LiP I and II are also capable of inducing demethylation, intramolecular addition, rearrangement, hydroxylation, and other reactions in their substrates, leading to substrate disintegration. However, in the prolonged absence of the substrate to be reduced, LiP II reacts with peroxide to form a LiP III compound, leading to irreversible inactivation of the enzyme. The role of veratryl alcohol, produced naturally by *P. chrysosporium*, would therefore be to preserve enzyme inactivation when there is no or no longer any substrate to oxidize in the medium [67],[68],[70],[71].

$$\text{Lip }\left[\text{Fe III}\right]+{\text{H}}_{2}{\text{O}}_{2}\to \text{LiP I }\left[\text{Fe IV}={\dot{O}}^{+}\right]+{\text{H}}_{2}\text{O}$$ (4)

$$\text{Lip I }\left[\text{Fe IV}={\dot{O}}^{+}\right]+\text{SH}\to \text{LiP II }\left[\text{Fe IV}=\text{O}\right]+{\dot{S}}^{+}$$ (5)

$$\text{Lip II }\left[\text{Fe IV}=\text{O}\right]+\text{SH}\to \text{LiP }\left[\text{Fe III}\right]+{\dot{S}}^{+}+{\text{H}}_{2}\text{O}$$ (6)

### 6.4. Laccases

Laccases are a large group of isoenzymes with molecular weights ranging from 50 to 100 kDa, partly because they can be highly glycosylated. The carbons in the side chains account for 10 to 40% of the enzyme's molecular weight. Glycosylation of laccases is thought to play a role in their extracellular secretion, thermal stability, and retention of copper atoms. Over a hundred laccases have been discovered. They are produced not only by fungi, but also by other organisms (plants, insects, bacteria, etc.). However, those produced by fungi have a higher redox potential (+800 mV) than those produced by insects and plants. Unlike the previous two enzymes, laccase does not catalyze reactions involving hydrogen peroxide [72]. They are “blue multicopper oxidases” and oxidize a wide range of aromatic and non-aromatic compounds by producing two molecules of water resulting from the reduction of two oxygen atoms. These substrates are transformed into free radicals depending on their structure and the reaction conditions. Laccase production tends to be low when the fungi evolve in a liquid rather than a solid medium. However, the concentration of aromatic compounds such as 2,5-xylidine seems to have a positive effect on laccase production. Laccases have 4 copper atoms in their structure. One of them is located at the T1 site. This atom, in its oxidized state, is responsible for the blue-green color of the medium. T1 is the binding site for the substrate to be reduced. The other three atoms are grouped at sites T2/T3. These sites catalyze the reduction of molecular oxygen to water. Laccases are not substrate-specific but are capable of oxidizing a wide variety of compounds. There are, however, differences in substrate preference from one isoenzyme to another. Among laccase substrates, we find ortho and paradiphenols, methoxy-substituted phenols, polyphenols, aromatic amines, benzenthiols, hydroxindols, 1-naphthol and syringaldazine [30],[72]. Laccase uses over 100 different mediators. ABTS (2,2′-azino-bis(3-ethylbenzothiazoline-6-sulfonic acid) and HBT (1-hydroxybenzotriazole) are the most commonly used. These mediators are low-molecular-weight compounds with a higher redox potential (>900 mV) than laccase. They are oxidized by the enzyme to give highly reactive radicals. The radicals formed can attack substrates with molecular weights too large to bind to the enzyme's catalytic sites. In the case of ABTS, laccase performs electron transfer to this mediator. The optimum temperature for laccases is between 30 and 60 °C. Laccase activity is inhibited in the presence of compounds capable of binding to the T2 and T3 sites without producing radicals, including metal ions and fatty acids.

## 7. Culture in solid fermentation

### 7.1. Inoculum preparation

Inoculum preparation usually begins with a concentrated suspension of spores obtained from a commercial retailer or on request from research organizations. It is also possible to prepare an inoculum after isolating the strain in its natural environment. The concentrated spore suspension is obtained by harvesting spores from the surface of a mycelium agar culture. Between the 3rd and 5th day of mycelium culture in a 100 mm × 15 mm petri dish, 20 mL of distilled water is poured onto the surface of the mycelium and gently scraped with a sterile rod to release the spores. The obtained suspension is then collected in a sterile tube and diluted to obtain a spore concentration close to 1 × 10^6^ spores/mL [73].

Spores can easily be counted using a microscope and a hemacytometer [74]. Determining the absorbance of the desired suspension can help to speed up the process in cases where it is necessary to prepare several suspensions at once, as spore counting can be a slow and tedious task [75]. Once the spore concentration of the suspension is achieved, 1 to 2 mL of the suspension is added per 100 mL of culture medium. The composition of the culture medium may vary from strain to strain and according to the strain supplier's instructions, but it always includes one or more sources of carbon, nitrogen, vitamins, and nutrients essential to the strain's primary metabolism, plus water. For example, for *P. chrysosporium* strain A-381 (ATCC 48746), the culture medium consists of (per 1000 mL) of yeast extract (3.0 g), malt extract (3.0 g), dextrose (10.0 g) and peptone (5.0 g) and water (1000 mL). For *P. chrysosporium* BKMF-1767 (CCTCC AF96007), the culture medium consists of glucose (10 g), of ammonium tartrate (0.206 g), KH_2_PO_4_ (2 g), MgSO_4_ (0. 5 g), FeSO_4_-7H_2_O (0.115 g), CaCl_2_ (0.1 g), ZnSO_4_-7H_2_O (0.089 g), CuSO_4_-5H_2_O (0.05 g), and vitamin B1 (0.001 g) dissolved in sodium tartrate buffer (20 mM, pH = 4.5). The culture medium is autoclaved at 121 °C for 20 min, then cooled before inoculation with the prepared spore suspension. This is then placed in a shaker incubator at 30 °C and 180 rpm for several days (3 to 5 days) [76].

### 7.2. Solid fermentation

Solid-state fermentation (SSF) is defined as the growth of microorganisms on a moist, solid substrate in the absence or near absence of free liquid (usually water). The substrate serves both as a growth medium and as a nutrient source. This is an old technique. Egyptian bread-making (2600 BC) and Asian Koji sauce are two of the oldest documented applications. It is also used in the production of enzymes, pigments, flavors, antibiotics, biosurfactants, enriched proteins, and organic acids [77]. For fungi cultivation, SSF is the type of fermentation that best approximates their natural environment. SSF for fungi cultivation has many advantages over submerged fermentation (SmF). It takes less volume because it uses less water for the same substrate. The risk of contamination disturbance is lower, thanks to the fungi's ability to grow in a heterogeneous environment. Oxygen transfer is maximized and optimized in stirred reactors. There is little or no effluent to treat at the end of fermentation. Little or no chemicals are used. Fermentation products are not diluted as in liquid fermentation, but rather concentrated. Energy consumption is low. In addition, operating costs for solid fermentation are significantly lower compared to liquid fermentation [78]. However, major difficulties yet remain challenges for solid-state fermentation, notably the transition from a small to a full-scale (industrial) operation. In SSF, heat transfer is provided by air and is therefore poor. As volume increases, substrate compaction also becomes a problem. Another drawback is the high risk of dead zones (where there is no activity) in the substrate, even with agitation. The design of bioreactors for solid-state fermentation remains one of the major challenges for scaling up the process to date, due to the poor heat and oxygen transfer. To date, there are bioreactors well known for their advantages, but which also suffer from drawbacks that often disqualify them from certain small or large-scale applications. Bioreactors are generally classified according to their aeration system. A distinction is made between free aeration bioreactors and forced aeration bioreactors. The former include laboratory glassware, plate chambers, rotating drums, and stirred drums, while the latter include fixed-bed and fluidized-bed reactors.

### 7.3. Bioreactors

#### 7.3.1. Laboratory glassware

**Figure 4.**
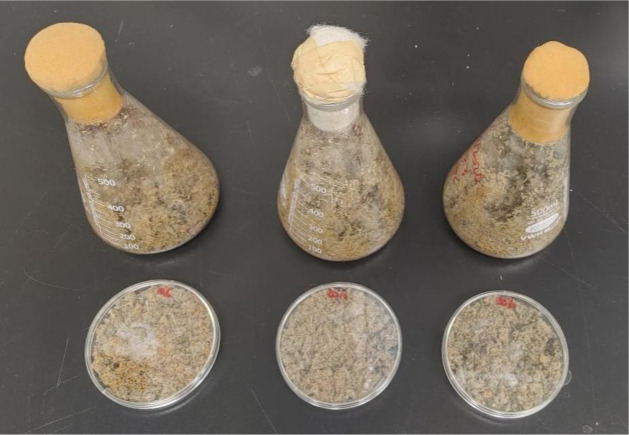
Solid state fermentation with *P. chrysosporium* on corn maize residues and black spruce chips in flasks and petri dishes.

At a laboratory scale, solid fermentation can be carried out in wide-mouth Erlenmeyer, jars or flasks [79]. These types of reactors have the advantage of being easily accessible, and solid fermentation is simple to operate. The biomass is introduced into the container and inoculated with the strain. The container can remain at room temperature or be kept in an incubator at the desired temperature (Figure 4). The pH can be adjusted according to fermentation conditions (water activity, etc.). Oxygen transfer and biomass homogeneity are improved by gentle mixing to avoid destroying the mycelium. Fermentation can be batch or fed-batch. In the latter case, the risk of contamination is higher. However, this type of fermentation is particularly suitable for screening fungi or biomass, or for rapidly testing cultivation conditions. The amount of substrate is small; in the gram range. Above a kilogram, the material becomes compacted, and aeration and oxygen transfer become a problem.

#### 7.3.2. Tray bed reactor-Incubator

**Figure 5.**
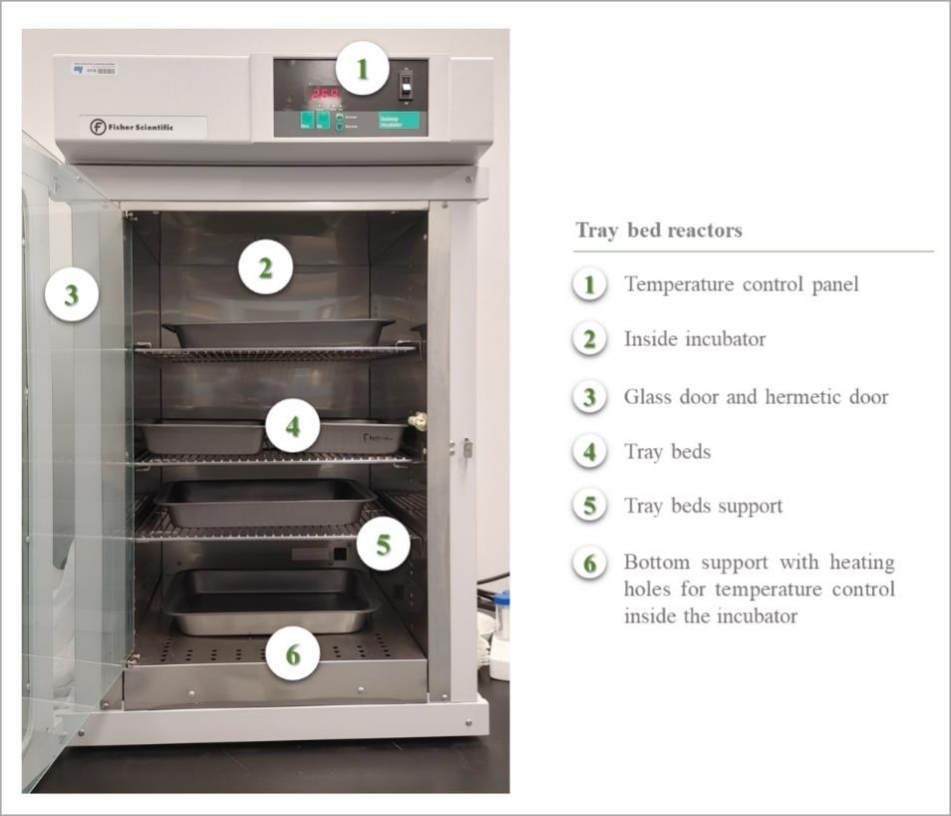
Tray bed reactors in an incubator.

The use of tray chambers goes back as far as the first traditional Koji fermentations in Asia. The biomass is laid out in layers (of varying thickness, between 5 and 15 cm) on perforated benches (also called trays) made of wood, aluminum, plastic, or other materials. The upper part of the biomass is in free contact with the air, while the lower part receives air through perforations in the benches. The benches are arranged on several levels and installed in an enclosure (reactor) or room (hall) in which the temperature is controlled (Figure 5) [80]. Tray chambers are well known and mastered as they present a rather basic design and because they have been used for a long time. However, heat accumulation in the biomass is one of the major drawbacks of this type of reactor. The biomass can be stirred manually from time to time, not only to dissipate the metabolic heat produced, but also to improve aeration. Otherwise, a significant temperature gradient is formed within the biomass [81]. Moreover, sometimes, the substrate concretes, causing mixing to be impossible. Several trays may be lost in this case. It is also possible to install a sprinkler system to control room humidity. The other major drawback with tray chambers is the space (volume, surface area) they require. It is an extensive technique. Scaling up involves increasing the number and/or surface area of trays [82].

#### 7.3.3. Fixed, rotating or stirred reactors

**Figure 6.**
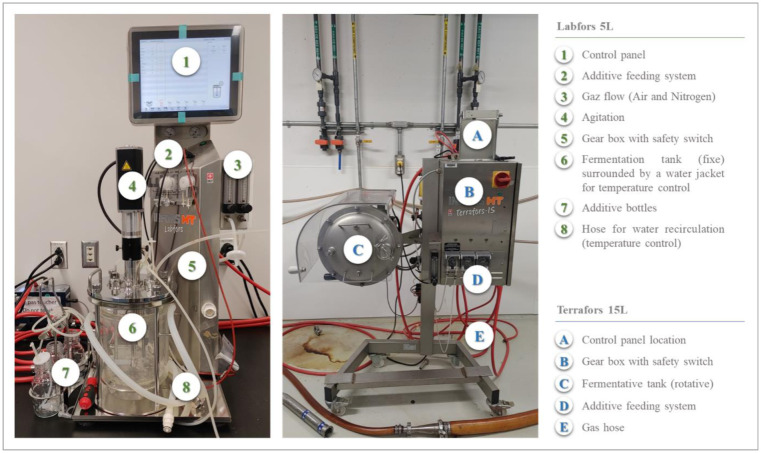
Fixe reactor LABFORS (5 L) and Rotative drum TERRAFORS (15 L).

Fixed reactors are vertical glass tanks specially designed for solid fermentations. They feature aeration, temperature control and aeration systems, and are generally equipped with a control panel (Figure 4). These reactors are available for small volumes (less than 10 L), because of the compaction problems that arise with larger volumes. Rotary or agitated drums, on the other hand, are better suited to large volumes. Rotary drums are horizontal (Figure 6) or vertical cylindrical enclosures half-filled with inoculated biomass and fitted with an air inlet and outlet. These drums rotate around an axis either mechanically or automatically, entraining the substrate in their movement. The drums are often fitted with fixed or mobile mixers (known as agitated rotary drums). The result is improved oxygen and CO_2_ transfer within the biomass [83]. Mycelium growth is faster and more uniform in drums than with tray chambers [84]. However, drum rotation speed has been shown to be inversely correlated with mycelium growth. Rotating drums also offer the advantage of batch, fed-batch or continuous operation. On the other hand, temperature control is a major challenge, even on a small scale [85].

#### 7.3.4. Fixed-bed reactor

The fixed-bed reactor is a column with a perforated bench or tray. Aeration is forced upwards through the perforations. The reactor is placed vertically, but can be horizontal or inclined [86]. The inoculated biomass is deposited in varying thicknesses on the bench. Temperature is controlled using a water bath in the laboratory model, or by means of a jacket of water maintained at the desired temperature and in direct contact with the reactor wall [87]. Humidity is maintained in the column by injecting water-saturated air. The major problems with this type of reactor are the risk of biomass compaction, the creation of privileged aeration channels within the biomass, the difficulty of removing heat when the biomass is very thick, and the pressure drop in the column; hence, the difficulty of using it on a large scale. On the other hand, in a scale model, forced aeration with agitation is quite effective in preventing heat build-up and pressure drop [82].

#### 7.3.5. Fluidized-bed reactor

The fluidized-bed reactor is similar to the vertical fixed-bed reactor, but with this type of reactor, biomass particles are kept in suspension thanks to a continuous flow of gas (from bottom to top). The flow must be just sufficient to prevent biomass particles from settling on the perforated bed or accumulating on the top of the reactor. In the former case, the perforations in the bed through which the air flows would clog up, and in the latter, the gas outlet would get obstructed. The fluidized-bed reactor is particularly suitable for small and light particles [86]. Occasionally, during operation, these particles agglomerate, fall and then accumulate on the perforated bench. An agitator can be installed in the reactor column not only to prevent particles from accumulating on the bench, but also to break down the agglomerates. In the laboratory model, i.e., for a volume of just a few liters, temperature control is as simple as in the fixed-bed reactor (water bath or water jacket), and oxygen transfer is maximal. The difficulty of fluidization and temperature control arises with larger volumes [88]. Particles are more likely to agglomerate. Using larger agitators would damage the mycelium, slowing down fungal growth and process efficiency.

### 7.4. Solid state fermentation parameters

#### 7.4.1. Water activity

Water activity (aw) is a dimensionless parameter. It reflects consistency in terms of the water content of a system, body, or matrix. In SSF, it is defined as the proportion of water available in a system for biochemical reactions. Water activity is, for a given temperature, the ratio of the system's vapor pressure to the reference vapor pressure; the reference pressure being that of pure distilled water, taken equal to 1 [89]. Every microorganism has a water activity threshold below which it is unable to maintain itself alive or even in a vegetative state. Most bacteria are unable to grow in environments with a water activity below 0.91. The threshold for fungi is 0.6 [90]. Water activity also influences the quality and spectrum of products that can be obtained. It changes throughout the fermentation process, depending on temperature, growth, aeration, agitation, and other conditions. Water plays three major roles in biological systems: (i) A structural role-i.e., it is involved in the constitution (composition) of cell walls; (ii) a solvent role, enabling biochemical reactions; and (iii) a transport role (heat and oxygen). The last two roles are an advantage for liquid fermentation and a challenge for solid fermentation. In solid fermentation, the continuity of the medium is ensured by the gas phase than the liquid (aqueous) phase as in liquid fermentation. As a result, there is a significant risk of temperature gradient formation, heterogeneity in biomass decomposition and aeration.

#### 7.4.2. Influence of pH

Controlling pH is a difficult operation in solid-state fermentation. Indeed, pH varies throughout fermentation and seems to be practically difficult to measure due to the absence of free water and the heterogeneity of the matrix (gas-liquid-solid) [91],[92]. The pH can only be measured locally and is therefore not representative of the medium as a whole. In semi-solid fermentation, on the other hand, pH measurement remains the same as in liquid fermentation, given the continuity of the medium provided by free water. In this case, pH becomes an input parameter. However, it is now well documented that fungi are tolerant of pH variation. Indeed, most fungi grow at pH levels between 4 and 9, but require a pH close to 7 for optimal sporulation [93],[94].

#### 7.4.3. Influence of temperature

Temperature is one of the most important parameters in solid fermentation. According to Manpreet et al. [95], during solid fermentation, 100 to 300 kJ of heat is released per kilogram of substrate. This heat comes mainly from the catabolic reactions of the micro-organisms, the synthesis of compounds used by their primary metabolism and the enzymatic reactions of lignin degradation [96]. The heat produced may vary significantly from one species to another, depending on growth rate or substrate type [97],[98]. However, in the absence of appropriate agitation and aeration systems, a temperature gradient is frequently created in the substrate. A gradient of 3 °C/cm thickness has been reported in a fermenter only 6.5 cm high [99]. Yet many white rot fungi used in solid fermentation have growth temperatures between 25 °C and 40 °C, and sometimes 50 °C for certain species [100]–[102]. However, this temperature range remains narrow in view of the metabolic heat released. Temperature control is therefore a key issue. This is one of the serious issues in reactor design. Manufactured bioreactors are now equipped with aeration and agitation systems, temperature sensors and a water jacket combined with a chiller to regulate the temperature inside the fermentation tank.

#### 7.4.4. Influence of aeration

The type of aeration is related to the choice of reactor. The gas composition and the aeration rate are crucial. Gas composition is a key factor in fermentation, especially in solid fermentation. Investigating different proportions of O_2_ and CO_2_ in the aeration gas, Villegas et al. [103] demonstrated that the qualitative (type of gas) and quantitative (relative proportion) composition of the gas has a strong influence on fungal growth, sugar consumption, pressure drop, and metabolic CO_2_ production. Indeed, the role of aeration is not only to supply oxygen, but also to regulate water activity (by water-saturated gas), to evacuate heat, CO_2_ and all the other volatile organic compounds produced by the metabolism of micro-organisms, which can inhibit biological activity once accumulated. Metabolic CO_2_, on the other hand, is characteristic of each species and reflects the growth rate of the mycelium. It is often used as an indicator of the stage of fermentation [104]. Aeration rate is another important factor to consider, particularly in forced aeration reactors. High aeration rates have been reported to destroy the mycelium and disrupt enzymatic activity. However, while it is clear that high levels are harmful, there are no general rules on the impact of moderate and low levels. For example, in the case of lactone production by solid fermentation, a very low aeration rate inhibits Acetyl-Coenzyme A activity and when the aeration rate increases, the enzyme's activity decreases [105]. In contrast, for lignolytic enzyme production with *Trametes versicolor*, Montoya et al. [106] tested all three aeration regimes: low, moderate and high. The highest laccase production was recorded under the lowest aeration rate. Thus, aeration must be optimized according to the microorganism, reactor type, fermentation conditions, and desired products.

#### 7.4.5. Influence of agitation

Substrate compaction and particle agglomeration limit fungi's access to the substrate. They also make heat evacuation difficult and limit the efficiency of aeration (oxygen transfer, etc.). The main role of agitation is to overcome these difficulties. Agitation can be continuous or intermittent (semi-continuous), and automatic or manual. Each of these modes has its advantages and disadvantages, and is highly dependent on the type of reactor, the type of substrate, the type of microorganism (mycelium) and the fermentation conditions. However, literature regularly mentions an optimum agitation speed of around 30 rpm. Agitation efficiency is not only dependent on agitation speed, but also on agitation duration. Vigorous agitation, however, even for a relatively short time, would lead to destruction of the mycelium and a reduction in enzyme production [92]. For forced aeration reactors, aeration can be a way of maintaining agitation. In pilot or industrial-scale fermenters, the limits of this practice quickly become apparent. Given the large substrate masses used in this type of fermenter, the risks of settling and particle agglomeration are much greater, and increasing the aeration flow is ineffective at the risk of causing the biomass to settle on the opposite wall to the direction of aeration.

#### 7.4.6. Fermentation duration

The duration of fermentation depends on the desired product (type and quantity), the capacity of the fungus(es), the inoculum (quantity), the substrate (type, quantity and quality) and the fermentation conditions (optimal or not). It can range from a few hours to several days or even months [107]–[109]. In general, metabolite production is low in the first few days and increases fairly rapidly until it reaches a production peak [110],[111]. Without monitoring, production slowly declines until it fades. The first part of the curve is directly correlated with the growth phase of the mycelium, while the decline in production is linked to various limiting factors such as the amount of nutrients available in the medium, the oxygen, and CO_2_ levels, and the presence and accumulation of inhibitors in the medium. Fermentation can continue as long as all optimal conditions are met.

## 8. Applications

*P. chrysosporium* has a wide range of applications which cannot be covered exhaustively in this review. The applications alone could be the subject of a dedicated review article. We present only four of the classic applications, namely biodelignification, decolorization, bioremediation of polyaromatic hydrocarbons and lignolytic enzyme production. However, the following table gives an overview of the diversity of *P. chrysosporium* applications.

**Table 1.** Application of *Phanerochate chrysosporium* for biodecomposition in diverse industries.

Industry	Substrate	Application	Conditions	Main result	References
Distillery industry	Vinasse (Colored recalcitrant wastewater)	Vinasse degradation	32 days, 39 °C	Decrease of phenolic concentration and color: 59.41%	Yuan et al. [12]
Textile dyeing industry	Wastewater	Decolorization	6 days, pH 4.5, 180 rpm, 39 °C, Glucose supplement	84% decolorization	Sangeeta et al.
Circuit printing industry	Copper from waste printed circuit	Leaching	14 days, 30 °C, 150 rpm	60.96 % leaching of copper	Lui et al. [112]
Drugs	Sulfamethazine (SMT) and Cadmium	Biotransformation	48 hours, 30 °C, 120 rpm	49% biotransformation for Cd and 62.9% for SMT	Guo et al. [113]
Fuel	Anthracene	Degradation	2 days, 37 °C, 80 rpm	Residual anthracene concentration was 16% at the end	Mohammadi and Nasernejad [114]
Textile industry	Azo dyes	Decolorization and detoxification	10 days, Concentration of 50 mg/L, 28 °C, 180 rpm, in the dark	82% Direct Yellow (DY27), 89% Reactive Black 5 (RB5), and 94% Reactive Red 120 (RR120) but increase of phytotoxicity	de Almeida et al. [115]
Papermill industry	Pentachlorophenol (PCP)	Removal	3 days, 0.23% lignisolfonate, 2% glucose suppelement	75% PCP removed	Aiken and Logan [116]
Agriculture	Olive mill effluent	Bioremediation	10 days, 135 rpm, 26 °C	59% soluble COD removal 70% colour removal 30% phenyl content removal	Diaz et al. [117]
Pulp and paper industry	Kraft Black Liquor effluent	Treatment	10 days, 25 °C, 150 rpm, Glucose supplement (3 g/L), pH 6	Removal of 65% COD, 37% colour and 56% phenolic compounds	Diaz et al. [118]
Civil and military industry	2,4,6-trinitrotoluene (TNT)	Removal	72 hours, 30 mg/L, 28 °C	82% removal	Ibbini et al. [119]
Plastic industry	Polylactic acid (PLA) Polystyrene (PS)	Degradation	35 days	34.35% degradation for PLA and 19.7% for PS	Wu et al. [120]
Textile industry	Dye	Decolorization	24 hours, 37 °C, Succinate buffer	100% decolorization of acid red 88, reactive black 5 and reactive orange 16	Ghasemi et al. [121]

### 8.1. Biodelignification

Biodelignification refers to the removal of lignin from lignocellulosic biomasses using microorganisms competent to perform this task. These are essentially white rot fungi (WRF). Biodignification was the very first application of *P. chrysosporium*. Today, the subject remains relevant, especially in the context of climate change and the exploration of sustainable alternatives to fossil fuels. Lignocellulosic biomass is a renewable source for bioethanol production, but it remains under-utilized. Global production of lignocellulosic biomass is estimated at 181.5 billion tons per year, of which only 8.2 billion are used, or just 4.5% billion tons [122]. Although the processes for converting cellulose into bioethanol are now well established, the difficulty with lignocellulosic biomass lies in the difficulty of accessing cellulose because of the lignin, which hinders recovery of this compound. To date, the delignification stage remains the limiting step in the valorization of lignocellulosic biomasses into bioethanol, due to the recalcitrance of lignin (J. Zhang et al., 2021). This step is estimated to account for 40% of bioethanol production costs with lignocellulosic biomasses [123]. Existing chemical, physical and physico-chemical methods often create more environmental issues than they solve. While many strategic advantages are associated with biodelignification, it is, however, criticized for the slowness of the process and problems of scale-up as highlighted above. Lignin is *P. chrysosporium*'s natural substrate, but the enzymatic system it secretes is effective only on destructured lignin or lignin with a relatively low molecular weight. Consequently, some authors suggest coupling biodignification with other treatment processes such as reactive extrusion [124]–[126]. Reactive extrusion is a fast, environmentally-friendly technique known for its ability to destructure the lignocellulosic complex, followed by an increase in the specific surface area of the particles. This preliminary action boosts the speed and yield of delignification by *P. chrysosporium*. Further research into pre-treatment methods involving biodelignification is required to make second-generation bioethanol production profitable on an industrial scale.

**Table 2.** Some results of biodelignification with *P. chrysosporium*.

Biomass	Particle	Reactor	Inoculum	Mass	Temp.	Time	Results	Reference
Apple pomace	20–60 mesh size	Petri dish	Spore suspension (1 mL)	5 g	28 °C	7 days	19.8 %delignification	Chen et al. [127]
Corn cobs	30 mesh size	Shake flask	Spore suspension (1×10^8^ spores/mL)	2.5 g	40 °C	3 days	78% delignification	Reddy et al. [128]
Coffee pulp	1–2 mm	-	Semi solid medium on biomass	-	35 °C	50 days	50.2% delignification	Phuong and Nguyen [129]
Paddy straw and pine needles	-	Bags	25 mL of water-washed mycelia	100 g	28 °C	28 days	Decrease of lignin from 27.11% to 14.11%	Gupta et al. [124]
Camelina straw and Switchgrass	10 mm	Vented bags	Malt extract broth	20 g	28 °C	35 days	24.08%, and 25.02% delignification for Camelina straw and switchgrass	Dao et al. [130]
Wheat straw	-	Flask	4 agar discs per flask	2.5 g	28 °C	15 days	45.6% delignification	Shrivastava and Sharma [131]
Olive pulp (25%) and wheat straw (75%)	2 to 5 mm for Olive pulp and 0.5 to 1.5 cm for wheat straw	Petri dishes	Colonized sorghum grains (1 g)	10 g	28 °C	84 days	60% delignification	Benaddou et al. [132]
Cotton stalks	Ground to pass through a 3 mm screen	Flask	Spore inoculation 1 ml (5 × 10^6^ spores/mL)	Solid loading was 5% w/v	39 °C	14 days	20.7% delignification	Shi et al. [133]
Switchgrass	1.6 mm	Vented bag	Malt extract broth (10 mL)	20 g	28 °C	21 days	23.6% delignification	Onu et al. [134]

### 8.2. Decolorization

There are over 100,000 different dyes on the market, for a production of around 700,000 tons per year [135],[136]. Most of this production ends up in polluted waters every year. The impact of colorants on the environment and human health is manifold. They are essentially complex and dangerous aromatic and heterocyclic compounds. Apart from the direct impact of this type of compound on human health (cancer, genetic mutation, coughing, diarrhea, eye and skin irritation, gastrointestinal infection, etc.), even at low concentrations (1 mg/L), these dyes affect water transparency, contribute a substantial organic load to the environment, significantly disrupt biological processes such as photosynthesis and destroy the survival conditions of organisms in their ecosystem [135],[137]. Moreover, they can undergo reactions with other molecules in the environment, resulting in even more dangerous compounds. Dyes found in the environment originate for the most part from the textile industry, but are also produced in a large number of industries such as cosmetics, ink production, pulp and paper, leather tanning, plastics and printing [135]. Yet dyes are persistent contaminants for which conventional water treatment processes are ineffective. Several chemical and physical methods have been developed, but remain either ineffective, energy-intensive, or expensive to deploy on an industrial scale. Because of their natural ability to degrade complex cyclic compounds, white rot fungi are being experimentally explored for the decolorization of industrial wastewater (usually from the textile industry) prior to discharge into the environment. Here again, *P. chrysosporium* is one of the fungi most frequently used in these processes. Some authors have reported promising results. Senthil et al. [138] highlighted the decolorization capacity of *P. chrysosporium* on azo dyes in submerged fermentation. When glucose, manganese, and ammonium salt were supplied at 0.5%, 0.1%, and 0.5% respectively, the rate of dye degradation by *P. chrysosporium* was close to 98% on the third day of culture. A few years earlier, Radha et al. [139] had investigated the efficiency of *P. chrysosporium* in stationary phase on seven other different types of dye (Methyl violet, Congo red, Acid orange, Acid red 114, Vat magenta, Methylene blue and Acid green) and showed that decolorization efficiency depended on the initial dye concentration. The best results (> 95%) were obtained with an initial concentration of 0.05g/L (35 °C, ph = 4.5). Similarly, for Gugel et al. [140], results were equally conclusive for the five dyes investigated, namely Orange II, Red 8BLP, Direct black 80, Direct yelllow 11, Basic brown 1. By the end of the seventh day of culture, all solutions containing the dyes appeared almost completely decolorized. Thus, *P. chrysosporium* is a promising decolorizing agent.

### 8.3. PAH Biomemediation

The concept of using *P. chrysosporium* in bioremediation is also based on the ability of the enzymatic system (including cytochrome P450 [141] of *P. chrysosporium* to mineralize polyaromatic hydrocarbons (PAHs), chlorinated aromatic hydrocarbons (CAHs), and a wide range of aromatic organopollutants, including pesticides, explosives, etc., into CO_2_ and H_2_O. Compounds in this group are known to pose significant environmental pollution problems [142]. For a long time, fungi were neglected in bioremediation in favor of bacteria, until their susceptibility to mineralize aromatic contaminants was highlighted by Bumpus et al. in their work on 22 different PAHs [6],[143]. Bacteria are often ineffective in environments saturated with aromatic compounds. PAHs originate mainly from anthropogenic sources, but can also come from natural sources (volcanic eruptions, bush fires, tree exudates, etc.) [144]. They come from the incomplete combustion of fossil fuels, exhaust smoke, tobacco smoke, etc. and are classified as Persistent Organic Pollutants (POPs) [145].

Their derivatives are equally or more toxic. A simple dehydration reaction can lead to carcinogenic or mutagenic compounds. PAHs are found in all matrices (soil, air and water). The efficiency of *P. chrysosporium* in mineralizing PAHs has been studied in a number of studies. Abo-state et al. [146] demonstrated that *P. chrysosporium* is able to completely (100%) or almost completely (>90%) degrade pyrene, acenaphthene, anthracene, fluoranthene, and phenanthrene in CO_2_ and H_2_O within seven (7) days at room temperature (25 °C), under different MnSO_4_ conditions (600 µM and 2000 µM) in agitated and non-agitated cultures. By comparing the ionization energies of 9 and 12 PAHs, respectively, Hammel and Kirk [147] and Bogan and Lamar [148] demonstrated that PAHs suitable for degradation by LiP from *P. chrysosporium* have ionization potentials of 7.55 ev or less. Wang et al. [149] identified LiP and MnP as the main enzymes in the *P. chrysosporium* system responsible for the degradation of PAHs in soils. These two enzymes were responsible for over 70% of phenanthrene degradation after 19 days of treatment. It has been identified that solubility is a key limiting factor for PAH degradation in soils. PAHs with low molecular weights are therefore easily degraded by *P. chrysosporium* than those with higher molecular weights [149],[150]. In aqueous media, phenanthrene and pyrene degradation approaches 100% in 60 days when the medium is nitrogen-poor and carbon-rich [151].

### 8.4. Production of lignolytic enzymes

Enzymes are valuable chemical compounds, especially in the food sector. According to Grand View Research, in 2021, the global enzyme market was worth 11.47 billion US dollars, and the forecast for 2028 is 17.88 billion, with an annual growth rate of 6.5% [152]. Amylases and then cellulases are the leading carbohydratases in demand to date [153]. With the development of second-generation biorefineries, there is a growing demand for lignolytic enzymes [154]. Lignolytic enzymes refer to enzymes capable of degrading the lignin contained in lignocellulosic biomasses, thereby making cellulose and hemicellulose accessible. There are three major types of ligninolytic enzymes: manganese peroxidase (MnP), lignin peroxidase (LnP) and laccase. Solid fermentation attempts to reproduce the natural (but controlled) environment in which white rot fungi naturally produce lignolytic enzymes. White rot fungi are grown in the presence of lignocellulosic material such as forest residues, agricultural residues, or any other type of lignocellulosic residue. Broadly the production of lignolytic enzymes under solid fermentation proceeds as follows: Biomass is sterilized and introduced into the bioreactor, then inoculated with either fungal spores or pre-cultured mycelium. Fungi grow under specific conditions (pH, temperature, agitation, etc.) depending on the fungi species, substrate type and bioreactor. A few hours or days after inoculation, the mycelium adsorbs and grows on biomass particles, degrading the lignin by means of lignolytic enzymes it produces. Lignin degradation is accompanied by the utilization of part of the sugars released (cellulose and hemicellulose) by the fungi to ensure their vital needs and growth (primary metabolism). The enzymes produced can then be recovered and purified for other applications. Enzyme production by solid fermentation has been proven to be in general more advantageous than liquid fermentation [155]. For example, Wang and Yang [81] reported that cellulase production in liquid media is $20/kg, whereas it is only $0.2/kg biomass in solid fermentation in comparison.

## 9. Perspectives

Considering the potential of *P. chrysosporium* to secrete powerful enzymes capable of degrading one of the most recalcitrant compounds in lignocellulosic biomasses; considering lignin to be the main cause of difficulties in the valorization of lignocellulosic biomasses for biorefineries, *P. chrysosporium* could play an important role in second-generation biorefineries by contributing to the biodelignification of lignocellulosic biomasses in order to increase their enzymatic digestibility during the enzymatic hydrolysis stage. Yet, in the specific context of lignocellulosic residues, given the rigidity of these biomasses, the use of *P. chrysosporium* on these residues should ideally be preceded by mechanical pre-treatment. Mechanical treatment should have the effect of disorganizing the structure of these biomasses and increasing the specific surface area of biomass particles to enable maximum efficiency of ligninolytic enzymes during the fermentation step. The challenges associated with this approach are manifold and include selecting the most suitable mechanical technology and its optimization without the formation of inhibitors; optimization of the conditions for growth and delignification of *P. chrysosporium* in solid or semi-solid fermentation; and minimization of pre-treatment costs to ensure the economic viability of the process.

## 10. Conclusions

*P. chrysosporium* has an excellent potential for a wide range of applications. The great interest in this fungus is linked to its ability to secrete several of the major enzymes involved in the natural degradation of lignin, but also of a broad range of aromatic compounds. Its importance is reinforced, on the one hand, by the fact that very few living organisms are capable of this exploit. On the other hand, *P. chrysosporium* is one of the few WRFs whose genome has been completely sequenced and has the advantage of being one of the most used WRF. Furthermore, given the development of bioengineering and the important role played by aromatic compounds in modern society (dyes, textiles, paint, food, energy, construction, automobiles, printing, pulp and paper, etc.), in contrast with their toxicity, and their increasingly alarming accumulation, the development of technologies using *P. chrysosporium* has just begun.

## Use of AI tools declaration

The authors declare they have not used Artificial Intelligence (AI) tools in the creation of this article.

## References

[1] Mohanty F, Swain SK, Oprea A.E., Grumezescu A.M. (2017). Chapter 18-Bionanocomposites for food packaging applications. Nanotechnology Applications in Food.

[2] Adane T, Adugna AT, Alemayehu E (2021). Textile industry effluent treatment techniques. J Chem.

[3] Kordbacheh F, Heidari G (2023). Water pollutants and approaches for their removal. Mater Chem Horiz.

[4] Anku WW, Mamo MA, Govender PP, Soto-Hernandez M., Palma-Tenango M., del Rosario Garcia-Mateos M. (2017). Phenolic compounds in water: sources, reactivity, toxicity and treatment methods. Phenolic compounds-natural sources, importance and applications.

[5] Pointing S (2001). Feasibility of bioremediation by white-rot fungi. Appl Microbiol Biotechnol.

[6] Bumpus JA (2021). White rot fungi and their potential use in soil bioremediation processes. Soil Biochem.

[7] Phanthong P, Reubroycharoen P, Hao X (2018). Nanocellulose: Extraction and application. Carbon Resour Convers.

[8] Zoghlami A, Paës G (2019). Lignocellulosic biomass: understanding recalcitrance and predicting hydrolysis. Front Chem.

[9] O'Neill MA, Moon RJ, York WS (2022). Glycans in bioenergy and materials science.

[10] He MQ, Zhao RL, Liu DM (2022). Species diversity of Basidiomycota. Fungal Diversity.

[11] Naranjo-Ortiz MA, Gabaldón T (2019). Fungal evolution: diversity, taxonomy and phylogeny of the Fungi. Biol Rev.

[12] Yuan Y, Bian LS, Wu YD (2023). Species diversity of pathogenic wood-rotting fungi (Agaricomycetes, Basidiomycota) in China. Mycology.

[13] Hibbett DS, Bauer R, Binder M, McLaughlin D.J., Spatafora J.W. (2014). 14 Agaricomycetes. Systematics and Evolution: Part A.

[14] Cao B, Haelewaters D, Schoutteten N (2021). Delimiting species in Basidiomycota: a review. Fungal Diversity.

[15] Miettinen O, Spirin V, Vlasák J (2016). Polypores and genus concepts in Phanerochaetaceae (Polyporales, Basidiomycota). MycoKeys.

[16] Corredor D, Duchicela J, Flores FJ (2024). Review of explosive contamination and bioremediation: insights from microbial and bio-omic approaches. Toxics.

[17] Mori T, Sugimoto S, Ishii S (2024). Biotransformation and detoxification of tetrabromobisphenol A by white-rot fungus Phanerochaete sordida YK-624. J Hazard Mater.

[18] Reddy CA, D'Souza TM (1994). Physiology and molecular biology of the lignin peroxidases of Phanerochaete chrysosporium. FEMS Microbiol Rev.

[19] Kato H, Takahashi Y, Suzuki H (2024). Identification and characterization of methoxy- and dimethoxyhydroquinone 1,2-dioxygenase from Phanerochaete chrysosporium. Appl Environ Microbiol.

[20] Martinez D, Larrondo LF, Putnam N (2004). Genome sequence of the lignocellulose degrading fungus Phanerochaete chrysosporium strain RP78. Nat Biotechnol.

[21] Kuppuraj SP, Venkidasamy B, Selvaraj D (2021). Comprehensive in silico and gene expression profiles of MnP family genes in Phanerochaete chrysosporium towards lignin biodegradation. Int Biodeterior Biodegrad.

[22] Gold MH, Alic M (1993). Molecular biology of the lignin-degrading basidiomycete Phanerochaete chrysosporium. Microbiol Rev.

[23] Coconi-Linares N, Ortiz-Vázquez E, Fernández F (2015). Recombinant expression of four oxidoreductases in Phanerochaete chrysosporium improves degradation of phenolic and non-phenolic substrates. J Biotechnol.

[24] Srinivasan C, Dsouza T, Boominathan K (1995). Demonstration of laccase in the white rot basidiomycete Phanerochaete chrysosporium BKM-F1767. Appl Environ Microbiol.

[25] Singh J, Das A, Yogalakshmi KN (2020). Enhanced laccase expression and azo dye decolourization during co-interaction of Trametes versicolor and Phanerochaete chrysosporium. SN Appl Sci.

[26] Rodríguez CS, Santoro R, Cameselle C (1997). Laccase production in semi-solid cultures of Phanerochaete chrysosporium. Biotechnol Lett.

[27] Thurston CF (1994). The structure and function of fungal laccases. Microbiology.

[28] Gnanamani A, Jayaprakashvel M, Arulmani M (2006). Effect of inducers and culturing processes on laccase synthesis in Phanerochaete chrysosporium NCIM 1197 and the constitutive expression of laccase isozymes. Enzyme Microb Technol.

[29] Dittmer JK, Patel NJ, Dhawale SW (1997). Production of multiple laccase isoforms by Phanerochaete chrysosporium grown under nutrient sufficiency. FEMS Microbiol Lett.

[30] Kunamneni A, Ballesteros A, Plou FJ, Yadav A.N., Mishra S., Singh S. (2007). Fungal laccase—a versatile enzyme for biotechnological applications. Communicating current research and educational topics and trends in applied microbiology.

[31] Coconi Linares N, Fernández F, Loske AM (2018). Enhanced delignification of lignocellulosic biomass by recombinant fungus Phanerochaete chrysosporium overexpressing laccases and peroxidases. J Mol Microbiol Biotechnol.

[32] Holzbaur ELF, Tien M (1988). Structure and regulation of a lignin peroxidase gene from Phanerochaete chrysosporium. Biochem Biophy Res Commun.

[33] Sarparast M, Dattmore D, Alan J (2020). Cytochrome P450 metabolism of polyunsaturated fatty acids and neurodegeneration. Nutrients.

[34] Matsuzaki F, Wariishi H (2004). Functional diversity of cytochrome P450s of the white-rot fungus Phanerochaete chrysosporium. Biochem Biophys Res Commun.

[35] Ning D, Wang H (2012). Involvement of Cytochrome P450 in Pentachlorophenol Transformation in a White Rot Fungus Phanerochaete chrysosporium. PLOS ONE.

[36] Ichinose H, Ukeba S, Kitaoka T (2022). Latent potentials of the white-rot basidiomycete Phanerochaete chrysosporium responsible for sesquiterpene metabolism: CYP5158A1 and CYP5144C8 decorate (E)-α-bisabolene. Enzyme Microb Technol.

[37] Ning D, Wang H, Zhuang Y (2010). Induction of functional cytochrome P450 and its involvement in degradation of benzoic acid by Phanerochaete chrysosporium. Biodegradation.

[38] Yadav J, Doddapaneni H, Subramanian V (2006). P450ome of the white rot fungus Phanerochaete chrysosporium: structure, evolution and regulation of expression of genomic P450 clusters. Biochem Soc Trans.

[39] Subramanian V, Yadav JS (2008). Regulation and heterologous expression of P450 enzyme system components of the white rot fungus Phanerochaete chrysosporium. Enzyme Microb Technol.

[40] Alic M, Kornegay Janet R, Pribnow D (1989). Transformation by complementation of an adenine auxotroph of the lignin-degrading basidiomycete phanerochaete chrysosporium. Appl Environ Microbiol.

[41] Schick Zapanta L, Hattori T, Rzetskaya M (1998). Cloning of phanerochaete chrysosporium leu2 by complementation of bacterial auxotrophs and transformation of fungal auxotrophs. Appl Environ Microbiol.

[42] Sharma KK, Gupta S, Kuhad RC (2006). Agrobacterium mediated delivery of marker genes to Phanerochaete chrysosporium mycelial pellets: a model transformation system for white-rot fungi. Biotechnol Appl Biochem.

[43] Suzuki H, MacDonald J, Syed K (2012). Comparative genomics of the white-rot fungi, Phanerochaete carnosa and P. chrysosporium, to elucidate the genetic basis of the distinct wood types they colonize. BMC Genomics.

[44] Lim YW, Baik KS, Chun JS (2007). Accurate delimitation of Phanerochaete chrysosporium and Phanerochaete sordida by specific PCR primers and cultural approach. J Microbiol Biotechnol.

[45] Vivekanandhan K, Ayyappadas MP, Abirami SKG (2021). Biodegradation of sago effluent by white- rot fungus Phanerochaete chrysosporium. Int Life Sci Pharma Res.

[46] Liu L, Qin Y, Li P (2016). Improvement in continuous cropping of cut chrysanthemum by phanerochaete chrysosporium. Pak J Bot.

[47] Dahiya J, Singh D, Nigam P (2001). Decolourisation of synthetic and spentwash melanoidins using the white-rot fungus Phanerochaete chrysosporium JAG-40. Bioresour Technol.

[48] Zhao J, de Koker TH, Janse BJH (1995). First report of the white rotting fungus Phanerochaete chrysosporium in South Africa. S Afr J Bot.

[49] Bin D, Yumei X, Hailong D (2019). *Phanerochaete chrysosporium* strain B-22, a parasitic fungus infecting *Meloidogyne incognita*. bioRxiv.

[50] Yang DQ (2005). Isolation of wood-inhabiting fungi from Canadian hardwood logs. Can J Microbiol.

[51] Khalil H, Legin E, Kurek B (2021). Morphological growth pattern of *Phanerochaete chrysosporium* cultivated on different Miscanthus x giganteus biomass fractions. BMC Microbiol.

[52] Liu L, Li H, Liu Y (2020). Whole transcriptome analysis provides insights into the molecular mechanisms of chlamydospore-like cell formation in *Phanerochaete chrysosporium*. Front Microbiol.

[53] Bánki O, Roskov Y, Döring M (2024). Catalogue of Life checklist (Version 2024-03-26). Catalogue of Life.

[54] Franco-Duarte R, Černáková L, Kadam S (2019). Advances in chemical and biological methods to identify microorganisms—from past to present. Microorganisms.

[55] Calvo-Flores FG, Dobado JA (2010). Lignin as renewable raw material. ChemSusChem.

[56] Liu Q, Luo L, Zheng L (2018). Lignins: biosynthesis and biological functions in plants. Int J Mol Sci.

[57] Konan D, Koffi E, Ndao A (2022). An overview of extrusion as a pretreatment method of lignocellulosic biomass. Energies.

[58] Rosa FM, Mota TF, Busso C (2024). Filamentous fungi as bioremediation agents of industrial effluents: a systematic review. Fermentation.

[59] Lundell TK, Mäkelä MR, de Vries RP (2014). Chapter eleven-genomics, lifestyles and future prospects of wood-decay and litter-decomposing basidiomycota. Adv Bot Res.

[60] Yang C, Qin J, Sun S (2024). Progress in developing methods for lignin depolymerization and elucidating the associated mechanisms. Eur Polym J.

[61] Singh D, Chen S (2008). The white-rot fungus *Phanerochaete chrysosporium*: conditions for the production of lignin-degrading enzymes. Appl Microbiol Biotechnol.

[62] Larrondo LF, Vicuña R, Cullen D, Arora D.K., Berka R.M. (2005). 14 - *Phanerochaete chrysosporium* genomics. Applied Mycology and Biotechnology.

[63] Glenn JK, Gold MH (2022). Reprint of: purification and characterization of an extracellular mn (ll)-dependent peroxidase from the lignin-degrading basidiomycete, phanerochaete chrysosporium. Arch Biochem Biophys.

[64] Hofrichter M (2002). Review: lignin conversion by manganese peroxidase (MnP). Enzyme Microb Technol.

[65] Sundaramoorthy M, Youngs HL, Gold MH (2005). High-resolution crystal structure of manganese peroxidase:  substrate and inhibitor complexes. Biochemistry.

[66] Emami E, Zolfaghari P, Golizadeh M (2020). Effects of stabilizers on sustainability, activity and decolorization performance of Manganese Peroxidase enzyme produced by *Phanerochaete chrysosporium*. J Environ Chemi Eng.

[67] Falade AO, Nwodo UU, Iweriebor BC (2017). Lignin peroxidase functionalities and prospective applications. MicrobiologyOpen.

[68] Pollegioni L, Tonin F, Rosini E (2015). Lignin-degrading enzymes. FEBS J.

[69] Koduri RS, Tien M (1994). Kinetic analysis of lignin peroxidase: explanation for the mediation phenomenon by veratryl alcohol. Biochemistry.

[70] Koduri RS, Tien M (1995). Oxidation of Guaiacol by Lignin Peroxidase: ROLE OF VERATRYL ALCOHOL (*). J Biol Chem.

[71] Singh S, Cheng G, Sathitsuksanoh N (2015). Comparison of different biomass pretreatment techniques and their impact on chemistry and structure. Front Energy Res.

[72] Rivera-Hoyos CM, Morales-Álvarez ED, Poutou-Piñales RA (2013). Fungal laccases. Fungal Biol Revi.

[73] Gao L, Huang D, Cheng M (2023). Effect of *Phanerochaete chrysosporium* inoculation on manganese passivation and microbial community succession during electrical manganese residue composting. Bioresour Technol.

[74] Zhang H, Xu X, Tan L (2021). The aggregation of Aspergillus spores and the impact on their inactivation by chlorine-based disinfectants. Water Res.

[75] Li N, Yu J, Wang X (2024). Growth, oxidative stress and ability to degrade tetrabromobisphenol a of *Phanerochaete chrysosporium* in the presence of different nano iron oxides. Water.

[76] Chen Z, Li N, Lan Q (2021). Laccase inducer Mn^2+^ inhibited the intracellular degradation of norfloxacin by Phanerochaete chrysosporium. Int Biodeterior Biodegrad.

[77] Mitchell DA, de Lima Luz LF, Krieger N (2011). Bioreactors for solid-state fermentation. Compr Biotechnol.

[78] Machado de Castro A, Fragoso dos Santos A, Kachrimanidou V, Pandey A., Larroche C., Soccol C.R. (2018). Chapter 10-Solid-state fermentation for the production of proteases and amylases and their application in nutrient medium production. Current Developments in Biotechnology and Bioengineering.

[79] Durand A (2003). Bioreactor designs for solid state fermentation. Biochem Eng J.

[80] Krishania M, Sindhu R, Binod P, Pandey A., Larroche C., Soccol C.R. (2018). Design of Bioreactors in Solid-State Fermentation. Current Developments in Biotechnology and Bioengineering.

[81] Wang L, Yang ST, Yang S.T. (2007). Chapter 18-Solid state fermentation and its applications. Bioprocessing for Value-Added Products from Renewable Resources.

[82] Arora S, Rani R, Ghosh S (2018). Bioreactors in solid state fermentation technology: Design, applications and engineering aspects. J Biotechnol.

[83] Webb C (2017). Design aspects of solid state fermentation as applied to microbial bioprocessing. J Appl Biotechnol Bioeng.

[84] Robinson T, Nigam P (2003). Bioreactor design for protein enrichment of agricultural residues by solid state fermentation. Biochem Eng J.

[85] Nava I, Favela-Torres E, Saucedo-Castaneda G (2011). Effect of mixing on the solid-state fermentation of coffee pulp with Aspergillus tamarii. Food Technol Biotechnol.

[86] Ge X, Vasco-Correa J, Li Y, Larroche C., Sanromán M.Á., Du G. (2017). 13-Solid-state fermentation bioreactors and fundamentals. Current Developments in Biotechnology and Bioengineering.

[87] Lonsane BK, Ghildyal NP, Budiatman S (1985). Engineering aspects of solid state fermentation. Enzyme Microb Technol.

[88] Werther J (2007). Fluidized-Bed Reactors. Ullmann's Encyclopedia Indust Chem.

[89] Fernández VM, Gargaud M., Amils R., Quintanilla J.C. (2011). Water activity. Encyclopedia of Astrobiology.

[90] Allen LV (2018). Quality control: water activity considerations for beyond-use dates. Int J Pharm Compd.

[91] do Nascimento FV, de Castro AM, Secchi AR (2021). Insights into media supplementation in solid-state fermentation of soybean hulls by *Yarrowia lipolytica*: Impact on lipase production in tray and insulated packed-bed bioreactors. Biochem Eng J.

[92] Teixeira MCV, Alves EH, de Oliveira FP (2019). Automation of solid state fermentation reactor for enzymes synthesis. Revista Univap.

[93] Sala A, Barrena R, Artola A (2019). Current developments in the production of fungal biological control agents by solid-state fermentation using organic solid waste. Crit Rev Environ Sci Technol.

[94] Suryadi H, Judono JJ, Putri MR (2022). Biodelignification of lignocellulose using ligninolytic enzymes from white-rot fungi. Heliyon.

[95] Manpreet S, Sawraj S, Sachin D (2005). Influence of process parameters on the production of metabolites in solid-state fermentation. Malay J Microbiol.

[96] Rosenberg E, Zilber-Rosenberg I (2016). Do microbiotas warm their hosts?. Gut Microbes.

[97] Wu ZD, Zhang Q, Yin J (2020). Interactions of mutiple biological fields in stored grain ecosystems. Sci Rep.

[98] Liu C, Chen G, Zhou Y (2022). Investigation on compression and mildew of mixed and separated maize. Food Sci Nutr.

[99] de Reu JC, Zwietering MH, Rombouts FM (1993). Temperature control in solid substrate fermentation through discontinuous rotation. Appl Microbiol Biotechnol.

[100] Azad K, Halim MA, Hossain F (2013). Optimization of culture conditions for the production of xylanase by two thermophilic fungi under solid state fermentation. J Asiat Soc Bangladesh Sci.

[101] Bhati P (2019). Effect of temperatures on the growth of floral waste degrading fungi. Fungal Territory.

[102] Ouahiba G, Yasmina S, Azzeddine B (2021). Optimization of endoglucanase production from Sarocladium kiliense strain BbV1 under solid state fermentation, using response surface methodology. PONTE Int Sci Res.

[103] Villegas E, Aubague S, Alcantara L (1993). Solid state fermentation: Acid protease production in controlled CO_2_ and O_2_ environments. Biotechnol Adv.

[104] Cruz-Cordova T, Roldán-Carrillo T, Dıaz-Cervantes D (1999). CO_2_ evolution and ligninolytic and proteolytic activities of Phanerochaete chrysosporium grown in solid state fermentation. Res Conserv Recycl.

[105] Try S (2018). Production d'arômes par fermentation en milieu solide: Université Bourgogne Franche-Comté.

[106] Montoya S, Patiño A, Sánchez ÓJ (2021). Production of lignocellulolytic enzymes and biomass of trametes versicolor from agro-industrial residues in a novel fixed-bed bioreactor with natural convection and forced aeration at pilot scale. Processes.

[107] Kwanga SN, Djuffo DT, Boum AT (2022). Effect of solid-state fermentation on the essential oil yield of curcuma longa residues. Waste Biomass Valorization.

[108] Mardawati E, Sinurat Y, Yuliana T (2020). Production of crude xylanase from *Trichoderma* sp. using reutealis trisperma exocarp substrate in solid state fermentation. IOP Conf Ser Earth Environ Sci.

[109] Naeimi S, Khosravi V, Varga A (2020). Screening of organic substrates for solid-state fermentation, viability and bioefficacy of *Trichoderma harzianum* AS12-2, a biocontrol strain against rice sheath blight disease. Agronomy.

[110] Tai WY, Tan JS, Lim V (2019). Comprehensive studies on optimization of cellulase and xylanase production by a local indigenous fungus strain via solid state fermentation using oil palm frond as substrate. Biotechnol Prog.

[111] Wu C, Zhang F, Li L (2018). Novel optimization strategy for tannase production through a modified solid-state fermentation system. Biotechnol Biof.

[112] Liu Q, Bai Jf, Gu WH (2020). Leaching of copper from waste printed circuit boards using Phanerochaete chrysosporium fungi. Hydrometallurgy.

[113] Guo X, Peng Z, Huang D (2018). Biotransformation of cadmium-sulfamethazine combined pollutant in aqueous environments: Phanerochaete chrysosporium bring cautious optimism. Chem Eng J.

[114] Mohammadi A, Nasernejad B (2009). Enzymatic degradation of anthracene by the white rot fungus Phanerochaete chrysosporium immobilized on sugarcane bagasse. J Hazard Mater.

[115] de Almeida AP, Macrae A, Ribeiro BD (2021). Decolorization and detoxification of different azo dyes by Phanerochaete chrysosporium ME-446 under submerged fermentation. Br J Microbiol.

[116] Aiken BS, Logan BE (1996). Degradation of pentachlorophenol by the white rot fungus Phanerochaete chrysosporium grown in ammonium lignosulphonate media. Biodegradation.

[117] Díaz AI, Ibañez M, Laca A (2021). Biodegradation of olive mill effluent by white-rot fungi. Appl Sci.

[118] Díaz AI, Laca A, Lima N (2022). Treatment of kraft black liquor using basidiomycete and ascomycete fungi. Process Saf Environ Prot.

[119] Ibbini J, Al-Kofahi S, Davis LC (2024). Investigating the potential of *Fusarium solani* and *Phanerochaete chrysosporium* in the removal of 2,4,6-TNT. Appl Biochem Biotechnol.

[120] Wu F, Guo Z, Cui K (2023). Insights into characteristics of white rot fungus during environmental plastics adhesion and degradation mechanism of plastics. J Hazard Mater.

[121] Ghasemi F, Tabandeh F, Bambai B (2010). Decolorization of different azo dyes by Phanerochaete chrysosporium RP78 under optimal condition. Int J Environ Sci Technol.

[122] Mujtaba M, Fernandes Fraceto L, Fazeli M (2023). Lignocellulosic biomass from agricultural waste to the circular economy: a review with focus on biofuels, biocomposites and bioplastics. J Cleaner Prod.

[123] Sindhu R, Binod P, Pandey A (2016). Biological pretreatment of lignocellulosic biomass--An overview. Bioresour Technol.

[124] Gupta A, Preetam A, Ghosh P (2023). A novel combinatorial approach for cleaner production of biodegradable sheets from the combination of paddy straw and pine needle waste. J Cleaner Prod.

[125] Gupta A, Tiwari A, Ghosh P (2023). Enhanced lignin degradation of paddy straw and pine needle biomass by combinatorial approach of chemical treatment and fungal enzymes for pulp making. Bioresour Technol.

[126] Konan D, Rodrigue D, Koffi E (2024). Combination of technologies for biomass pretreatment: a focus on extrusion. Waste Biomass Valorization.

[127] Chen J, Zhou J, Yuan R (2024). Mild pretreatment combined with fed-batch strategy to improve the enzymatic efficiency of apple pomace at high-solids content. BioEnergy Res.

[128] Reddy KT, Kocher GS, Singh A (2024). Pretreatment and saccharification of corn cobs using partially purified fungal ligninozymes. Biofuels Bioprod Bioref.

[129] Phuong D, Nguyen L (2023). Coffee pulp pretreatment methods: A comparative analysis of hydrolysis efficiency. Foods Raw Mater.

[130] Dao CN, Tabil LG, Mupondwa E (2023). Microbial pretreatment of camelina straw and switchgrass by Trametes versicolor and Phanerochaete chrysosporium to improve physical quality and enhance enzymatic digestibility of solid biofuel pellets. Renewable Energy.

[131] Shrivastava A, Sharma RK (2023). Conversion of lignocellulosic biomass: Production of bioethanol and bioelectricity using wheat straw hydrolysate in electrochemical bioreactor. Heliyon.

[132] Benaddou M, Hajjaj H, Diouri M (2023). Fungal treatment and wheat straw blend for enhanced animal feed from olive pulp. J Ecolog Eng.

[133] Shi J, Sharma-Shivappa RR, Chinn MS (2009). Microbial pretreatment of cotton stalks by submerged cultivation of Phanerochaete chrysosporium. Bioresour Technol.

[134] Onu Olughu O, Tabil LG, Dumonceaux T (2022). Optimization of solid-state fermentation of switchgrass using white-rot fungi for biofuel production. Fuels.

[135] Kalra A, Gupta A (2021). Recent advances in decolourization of dyes using iron nanoparticles: A mini review. Mater Today Proc.

[136] McMullan G, Meehan C, Conneely A (2001). Microbial decolourisation and degradation of textile dyes. Appl Microbiol Biotechnol.

[137] Ganaie RJ, Rafiq S, Sharma A (2023). Recent advances in physico-chemical methods for removal of dye from wastewater. IOP Conf Ser Earth Environ Sci.

[138] Senthilkumar S, Perumalsamy M, Janardhana Prabhu H (2014). Decolourization potential of white-rot fungus Phanerochaete chrysosporium on synthetic dye bath effluent containing Amido black 10B. J Saudi Chem Soc.

[139] Radha KV, Regupathi I, Arunagiri A (2005). Decolorization studies of synthetic dyes using Phanerochaete chrysosporium and their kinetics. Process Biochem.

[140] Gugel I, Summa D, Costa S (2024). Mycoremediation of synthetic azo dyes by white-rot fungi grown on diary waste: a step toward sustainable and circular bioeconomy. Fermentation.

[141] Li Q, Wang J, Wang Z (2023). Surfactants double the biodegradation rate of persistent polycyclic aromatic hydrocarbons (PAHs) by a white-rot fungus Phanerochaete sordida. Environ Earth Sci.

[142] Fulekar MH, Pathak B, Fulekar J, Goltapeh E.M., Danesh Y.R., Varma A. (2013). Bioremediation of organic pollutants using *Phanerochaete chrysosporium*. Fungi as Bioremediators.

[143] Bumpus JA (1989). Biodegradation of polycyclic hydrocarbons by *Phanerochaete chrysosporium*. Appl Environ Microbiol.

[144] Lee AH, Lee H, Heo YM (2020). A proposed stepwise screening framework for the selection of polycyclic aromatic hydrocarbon (PAH)-degrading white rot fungi. Bioprocess Biosys Eng.

[145] Venkatraman G, Giribabu N, Mohan PS (2024). Environmental impact and human health effects of polycyclic aromatic hydrocarbons and remedial strategies: A detailed review. Chemosphere.

[146] Abo-State MAM, Osman ME, Khattab OH (2021). Degradative pathways of polycyclic aromatic hydrocarbons (PAHs) by *Phanerochaete chrysosporium* under optimum conditions. J Radi Res Appl Sci.

[147] Hammel KE, Kalyanaraman B, Kirk TK (1986). Oxidation of polycyclic aromatic hydrocarbons and dibenzo[p]-dioxins by Phanerochaete chrysosporium ligninase. J Biol Chem.

[148] Bogan BW, Lamar RT (1995). One-electron oxidation in the degradation of creosote polycyclic aromatic hydrocarbons by Phanerochaete chrysosporium. Appl Environ Microbiol.

[149] Wang C, Sun H, Li J (2009). Enzyme activities during degradation of polycyclic aromatic hydrocarbons by white rot fungus Phanerochaete chrysosporium in soils. Chemosphere.

[150] Zheng Z, Obbard JP (2002). Oxidation of polycyclic aromatic hydrocarbons (PAH) by the white rot fungus, Phanerochaete chrysosporium. Enzyme Microb Technol.

[151] Ding J, Chen B, Zhu L (2013). Biosorption and biodegradation of polycyclic aromatic hydrocarbons by Phanerochaete chrysosporium in aqueous solution. Chin Sci Bull.

[152] Research GV (2021). Enzymes Market Size, Share & Trends Analysis Report By Type (Industrial, Specialty), By Product (Carbohydrase, Proteases), By Source (Microorganisms, Animals), By Region, And Segment Forecasts, 2021 - 2028. Grand View Research 978-1-68038-022-4 978-1-68038-022-4. 153 p.

[153] Siqueira JGW, Rodrigues C, Vandenberghe LPdS (2020). Current advances in on-site cellulase production and application on lignocellulosic biomass conversion to biofuels: A review. Biomass Bioenergy.

[154] Niladevi KN, Singh nee' Nigam P., Pandey A. (2009). Ligninolytic enzymes. Biotechnology for Agro-Industrial Residues Utilisation: Utilisation of Agro-Residues.

[155] Xu FJ, Chen HZ, Li ZH (2001). Solid-state production of lignin peroxidase (LiP) and manganese peroxidase (MnP) by Phanerochaete chrysosporium using steam-exploded straw as substrate. Bioresour Technol.

